# Pneumopathie interstitielle aiguë fatale sous docetaxel: à propos d'un cas et revue de la littérature

**DOI:** 10.11604/pamj.2016.24.119.8902

**Published:** 2016-06-07

**Authors:** Sami Aziz Brahmi, Seddik Youssef, Fatima Zahra Ziani, Said Afqir

**Affiliations:** 1 Service d'Oncologie Médicale, Centre Hospitalier Mohammed VI, Oujda, Maroc; 2 Service d'Oncologie Médicale, Centre Hospitalier, Hassan II, Fès, Maroc

**Keywords:** Cancer, pneumopathie interstitielle, docetaxel, Cancer, interstitial lung disease, docetaxel

## Abstract

Le docetaxel est un agent de chimiothérapie appartenant à la famille des taxanes. Ce médicament est largement utilisé dans le traitement des cancers. La pneumopathie intestitielle est une toxicité rare mais redoutable du fait d'un risque de mortalité élevé. On rapporte un cas d'une patiente atteinte d'un cancer du sein qui a présenté une pneumopathie interstitielle aigue fatale à la suite d'une chimiothérapie adjuvante par docetaxel. Le clinicien doit être conscient de ce risque et doit le considérer comme un diagnostique différentiel en cas de symptômes respiratoire chez un patient recevant du docetaxel.

## Abstract (en)

Docetaxel is a chemotherapeutic agent belonging to the taxane family. This drug is widely used to treat cancers. Interstitial lung disease is a rare but serious toxicity due to the high mortality risk. We report a case of a patient with breast cancer who had fatal acute interstitial lung disease after auxiliary chemotherapy with docetaxel. The clinician should be aware of this risk and should consider it in differential diagnosis in patients with respiratory symptoms treated with docetaxel.

## Introduction

Le docetaxel est un agent de chimiothérapie appartenant à la famille des taxanes. Ce médicament est largement utilisé dans le traitement des cancers. Actuellement, il est indiqué dans le cancer du sein, le cancer du poumon non petites cellules, les cancers de la tête et du cou et le cancer de la prostate. Ces principaux effets secondaires sont: la myélosupression, l'alopécie, la fatigue, les réactions d'hypersensibilité, les neuropathies périphériques, et la rétention hydrique. La pneumopathie intestitielle est une toxicité rare mais redoutable du fait d'un risque de mortalité élevé [1]. On rapporte un cas d'une patiente atteinte d'un cancer du sein qui a présenté une pneumopathie interstitielle aiguë fatale à la suite d'une chimiothérapie par docetaxel.

## Patient et observation

**Figure 1:**
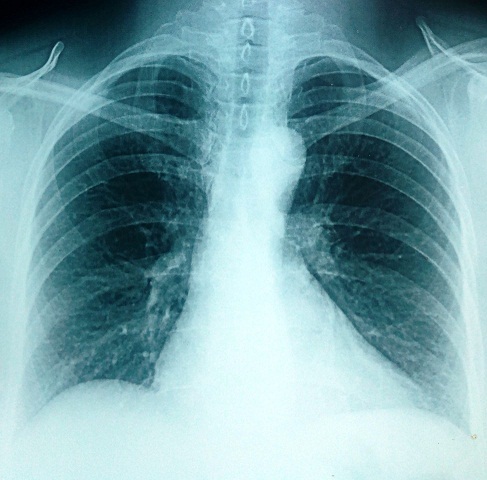
Radiographie thoracique montrant un syndrome alvéolo-interstitiel diffu bilatéral

**Figure 2:**
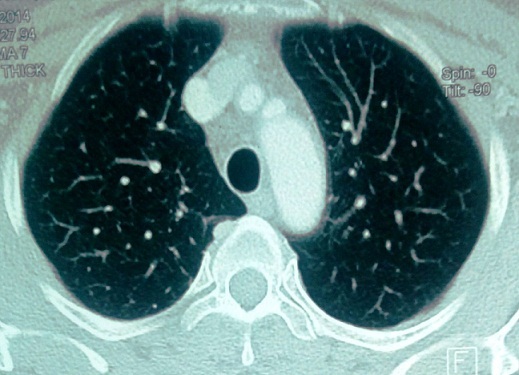
Coupe scannographique thoracique montrant un infiltrat interstitiel diffu bilatéral

Il s'agit d'une patiente de 35 ans, sans antécédents pathologiques notables notamment pas de terrain allergique ni de pneumopathie. La patiente a été admise dans notre service pour chimiothérapie adjuvante d'un cancer du sein. Elle avait eu une tumorectomie gauche avec curage axillaire pour une lésion du sein gauche classé ACRV. Il s'agissait d'un carcinome canalaire infiltrant grade III de Scarff Bloom Richardson avec présence d'embols vasculaires. La tumeur a été classée pT2N2MX avec une taille tumorale de 4 cm et atteinte de 6 ganglions sur 14. A l'examen immunohistochimique il s'agissait dune tumeur triple négative sans expression des récepteurs hormonaux ni de l'HER2 (human epithelial receptor 2). Le bilan d'extension fait d'un scanner thoraco-abdominopelvien et d'une scintigraphie osseuse a été normal. L'examen clinique à son admission trouvait une patiente OMS à 0 avec une cicatrice de tumorectomie propre sans autre anomalies. Une indication d'une chimiothérapie séquentielle adjuvante a été retenue suivie d'une radiothérapie adjuvante. La patiente a reçu 3 cure à base d'AC60 (adriamycine 60mg/m^2^, cyclophosphamide 600mg/m^2^) chaque 21 jours avec une bonne tolérance. Puis 3 cures de docetaxel ont été a programmé à une dose de 100mg/m^2^ chaque 21 jours. Une prémédication par corticoïdes et antihistaminiques a précédée chaque cure de docetaxel. La tolérance des deux premières cures a été bonne. Onze jours après la troisième cure de docetaxel, la patiente s'est présentée en consultation avec une dyspnée. A l'examen clinique elle était polypnéique avec une fréquence respiratoire à 30 cycles par minutes, normotendue et apyrétique avec une saturation de l'O2 à l'air ambiant à 60%. Une radiographie thoracique a été réalisée en urgence montrant un syndrome alvéolo-interstitiel diffu bilatéral (Figure 1). Un scanner thoracique avec injection de produit de contraste a été réalisé montrant un infiltrat interstitiel diffu des deux poumons sans signes d'embolie pulmonaire (Figure 2). La patiente a été admise en réanimation, mise sous oxygénothérapie, sous corticothérapie avec des bolus de méthylprednisolone à raison de 120mg deux fois par jour et une antibiothérapie empirique par tazocilline a été instauré. Le bilan biologique fait d'une numération formule sanguine était normal (hémoglobine=12g/dl, globules blancs=8540/mm^3^, plaquettes=220000/mm^3^). La CRP était à 200mg/l, le taux de pro calcitonine était à 0.2ng/ml. Le reste du bilan biologique notamment la fonction rénal, l'ionogramme et le bilan hépatique était normal. Une échographie cardiaque a montré une fonction cardiaque normale estimée à 64%, sans anomalies. La patiente a rapidement présenté une aggravation de son état respiratoire avec majoration de la dyspnée et de l'hypoxie nécessitant son intubation trois jours après son admission en réanimation. Elle a été mise sous antibiothérapie empirique et corticothérapie. La patiente est décédée une semaine après son admission en réanimation par défaillance respiratoire.

## Discussion

Les pneumopathies interstitielles sont une toxicité rare des taxanes. Avant de conclure à une toxicité pulmonaire du à ce médicament, il faut exclure les autres causes de pneumomathies interstitielles: infectieuses, d'origine cardiaque ou secondaires à une lymphangite carcinomateuse. Dans le cas de notre patiente l'origine infectieuse ou métastatique a été exclue, et la fonction cardiaque était normale à la suite d'un examen échographique. Bien que n′étant pas rapportés dans des observations cliniques, plusieurs cas de pneumopathies grade 4 ont été enregistrés dans des phases II évaluant le docetaxel en monothérapie [2, 3]. Cette toxicité se manifestait par un tableau de pneumopathie inexpliqué, fièvre, pneumonie bilatérale et hypoxie sévère ou une pneumopathie interstielle. Le docetaxel semble plus incriminé dans la survenue de pneumopathies sévères que la paclitaxel [4]. Une trentaine de cas de pneumopathies sévères secondaires aux taxanes ont été rapportés dans la littérature dont les deux tiers du au docetaxel [5]. Les patients traités par docetaxel ont développé une pneumopathie interstitielle quelques semaines après la cure de façon aiguë. Le mécanisme de survenue de la pneumopathie provoquée par le docetaxel demeure inconnu. Plusieurs hypothèses ont été proposées. Une première hypothèse stipule que le docetaxel peut entraîner une prolifération des cellules T cytotoxiques spécifiques dirigés contre un antigène pulmonaire co-exprimés par la tumeur, ce qui conduit à des lésions pulmonaire de type allergique 6. Une autre hypothèse dit que le docetaxel peut entraîner des lésions pulmonaires de façon directe par l'intermédiaire de ces métabolites [6]. Néanmoins le mécanisme immuno-allergique semble le plus plausible. Ce mécanisme est conforté par les résultats d'histologie de biopsie pulmonaire. En effet les résultats de biopsie de cas ont montré un infiltrat de cellules immunitaires au niveau de la muqueuse pulmonaire [7, 8]. Les modalités de prise en charge de cette toxicité sont inconnues. Dans la plupart des cas rapportés, la prise en charge est empirique et repose sur une corticothérapie à haute doses, une antibiothérapie à large spectre, une oxygénothérapie avec parfois une ventillation assistée. Selon une série de 31 cas de pneumopathie interstitielle induite par les taxanes, 35% des patients ont nécessité une ventilation assistée et le taux global de mortalité était de 42%, alors que le taux de mortalité chez les patients intubés était de 82% [5].

## Conclusion

Le docetaxel est une drogue de chimiothérapie largement utilisée dans le traitement du cancer du sein et d'autres cancers. Les patients qui reçoivent cette chimiothérapie présentent un risque de développer une pneumopathie interstitielle fatale. Le clinicien doit être conscient de ce risque et doit le considérer comme un diagnostique différentiel en cas de symptômes respiratoire chez un patient recevant du docetaxel.
